# Enhanced BMP Signaling Alters Human β‐Cell Identity and Function

**DOI:** 10.1002/adbi.202400470

**Published:** 2024-11-05

**Authors:** Esmée Dekker, Javier Triñanes, Amadeo Muñoz Garcia, Natascha de Graaf, Eelco de Koning, Françoise Carlotti

**Affiliations:** ^1^ Department of Internal Medicine Leiden University Medical Center Albinusdreef 2 Leiden 2333 ZA The Netherlands

**Keywords:** BMP2, BMP4, diabetes, human pancreatic islets, inflammation

## Abstract

Inflammation contributes to the pathophysiology of diabetes. Identifying signaling pathways involved in pancreatic β‐cell failure and identity loss can give insight into novel potential treatment strategies to prevent the loss of functional β‐cell mass in diabetes. It is reported earlier that the immunosuppressive drug tacrolimus has a detrimental effect on human β‐cell identity and function by activating bone morphogenetic protein (BMP) signaling. Here it is hypothesized that enhanced BMP signaling plays a role in inflammation‐induced β‐cell failure. Single‐cell transcriptomics analyses of primary human islets reveal that IL‐1β+IFNγ and IFNα treatment activated BMP signaling in β‐cells. These findings are validated by qPCR. Furthermore, enhanced BMP signaling with recombinant BMP2 or 4 triggers a reduced expression of key β‐cell maturity genes, associated with increased ER stress, and impaired β‐cell function. Altogether, these results indicate that inflammation‐activated BMP signaling is detrimental to pancreatic β‐cells and that BMP‐signaling can be a target to preserve β‐cell identity and function in a pro‐inflammatory environment.

## Graphical abstract

This study evaluates the association between bone morphogenetic protein (BMP)‐signaling and inflammation‐induced β‐cell failure in the context of diabetes. Results show that proinflammatory cytokines activate BMP signaling in human islets. Furthermore, enhanced BMP signaling reduces β‐cell marker expression, increases ER stress, and impairs β‐cell function. These results show that inflammation‐activated BMP signaling is detrimental to pancreatic β‐cells.

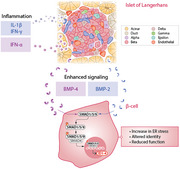

## 1 Introduction

Diabetes mellitus is characterized by the progressive loss of β‐cell mass and function, leading to chronic high blood sugar levels.^[^ ^1^ ^]^ Inflammation plays a role in the pathophysiology of both type 1 (T1DM) and type 2 (T2DM) diabetes mellitus, by altering β‐cell viability and function.^[^ ^2^, ^3^, ^4^, ^5^, ^6^, ^7^ ^]^ Distinct inflammatory mediators have been identified. Interferon α (IFNα) is a key component of inflammation during the early stages of T1DM,^[^ ^6^ ^]^ while the pro‐inflammatory cytokines IL1β (Interleukin 1β) and IFNγ (Interferon γ) released by infiltrated mononuclear cells in pancreatic islets are involved later in the development of the disease.^[^ ^7^ ^]^ Various studies describe islet infiltration by macrophages and elevated levels of cytokines, including IL1β, in type 2 diabetes.^[^ ^8^, ^9^, ^10^ ^]^

We previously reported that the immunosuppressive drug tacrolimus has a detrimental effect on human β‐cell identity and function by activating bone morphogenetic protein (BMP) signaling.^[^ ^11^ ^]^ BMP signaling has also been shown to contribute to the negative effects of inflammatory processes, such as in the pathophysiology of vascular endothelial cells^[^ ^12^, ^13^ ^]^ and synoviocytes in the context of rheumatoid arthritis.^[^ ^14^, ^15^ ^]^ In the context of pancreatic islets, inflammation has been reported to induce BMP2 expression.^[^ ^16^ ^]^

BMPs are members of the transforming growth factor (TGF‐β) family of genes that are involved in the control of a variety of biological processes including proliferation, differentiation, development, and homeostasis of tissues.^[^ ^17^ ^]^ Animal studies have shown that during pancreatic development, BMP signaling has a context‐dependent role.^[^ ^18^ ^]^ Although BMP4 stimulates pancreatic progenitor growth and maturation, endocrine differentiation is dependent on the inhibition of BMP signaling.^[^ ^19^, ^20^, ^21^ ^]^ Besides, BMP signaling is suppressed during the different stages of β‐cell development, as evidenced in protocols to differentiate human pluripotent stem cells toward the islet lineage.^[^ ^18^, ^22^ ^]^ Although administration of BMP2 and BMP4 has been reported to affect insulin secretion in rodent cells,^[^ ^23^, ^24^ ^]^ the function of BMP signaling in adult human β‐cells is largely unknown.

BMPs act through binding BMP type‐1 and type‐2 receptors. This complex activates BMP signaling mediators Smad‐1/5/9 by phosphorylation. Among the various BMP ligands, BMP2 and BMP4 have been described to be expressed in the human pancreas,^[^ ^25^, ^26^, ^27^ ^]^ and upregulated in the vasculature of animal models for T1DM and T2DM.^[^ ^28^, ^29^ ^]^ Furthermore, BMP2 and BMP4 are linked to beta cell dysfunction, obesity, and T2DM.^[^ ^30^, ^31^, ^32^ ^]^ BMP2 and BMP4 diverge from a common ancestral gene and their protein sequences are highly similar.^[^ ^33^ ^]^

In this study, we hypothesized that BMP signaling is enhanced upon inflammation, and may be responsible for some of the detrimental effects of inflammation‐induced β‐cell failure in diabetes.

## 2 Experimental Section

### 2.1 Single‐Cell RNA Sequencing

**Figure 1:**
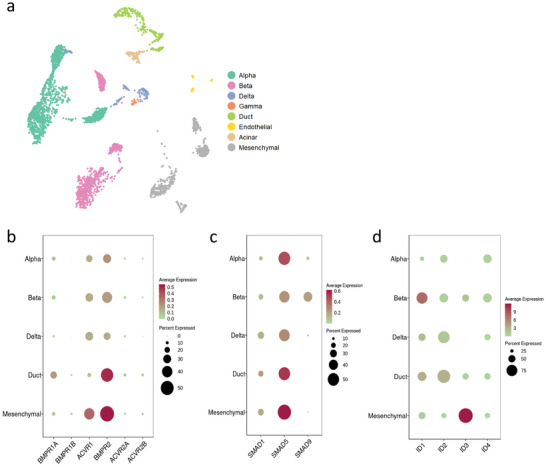
BMP signaling is active in human pancreatic islets. a) UMAP of different cell‐type clusters of isolated primary human islets. b) BMP type‐1 (*BMPR1A, BMPR1B, ACVR1*) and type‐2 (*BMPR2, ACVR2A* and *ACVR2B*) receptor expression in different cell clusters. c) Expression of BMP signaling mediators *SMAD1/5/9* in human pancreatic cells. d) Expression of BMP target genes *ID1‐4* in different cell types.

**Figure 2:**
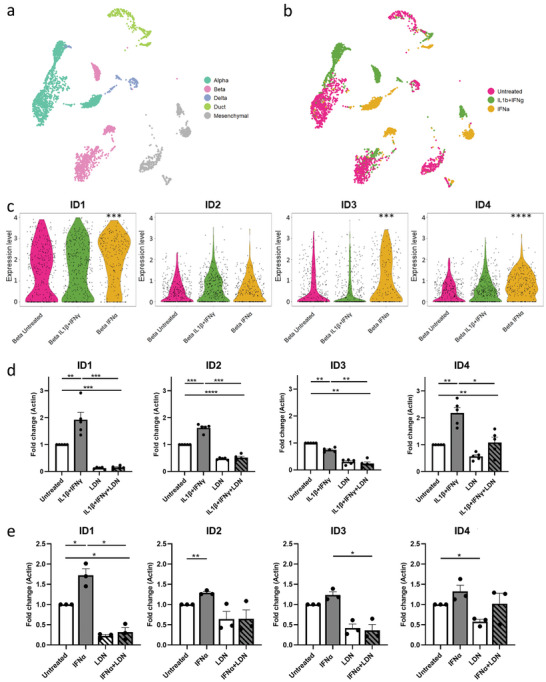
BMP signaling is enhanced in primary human islets upon inflammation. a) UMAP of different cell‐type clusters of isolated primary human islets treated with IL‐1β (1 ng mL⁻^1^) and IFNγ (50 ng mL⁻^1^), IFNα (1000 U mL⁻^1^), or untreated. b) UMAP of different treatment groups (untreated, IL‐1β and IFNγ, IFNα) of isolated primary human islets. c) Single‐cell RNA sequence gene expression of BMP target genes *ID1‐4* in untreated β ‐cells, IL‐1β and IFNγ treated β‐cells, and IFNα treated β‐cells. d) *ID*‐gene expression in primary human islets after 24 h treatment with IL‐1β and IFNγ and/or LDN (120 nM) normalized to the untreated control (n = 5). e) *ID*‐gene expression in primary human islets after 24 h treatment with IFNα and/or LDN (120 nM) (n = 3). ****p < 0.0001, ***p < 0.001, **p < 0.01, *p < 0.05.

The single cell‐RNA sequencing dataset originating from isolated human pancreatic islets treated or not with various stressors was analyzed. This single cell RNA sequence dataset was available from the Gene Expression Omnibus (GEO) with accession number GSE218316. The dataset was processed and analyzed as described.^[^ ^34^ ^]^ Cell clusters with less than 55 cells were not used in further analyses. **Figure** **1**, Figures S1 and S2 (Supporting Information) have been generated with data from the “untreated” condition only. In **Figure** **2**, the following conditions were compared using a Wilcoxon rank‐sum test: untreated control, cells treated with IL‐1β (1 ng mL⁻^1^; R&D Systems, Dublin, Ireland) and IFNγ (50 ng mL⁻^1^, R&D Systems, Dublin, Ireland), or treated with IFNα (1000 U mL⁻^1^, R&D Systems, Dublin, Ireland).

### 2.2 Cell Culture

Human islets were isolated from pancreases of cadaveric donors through the Eurotransplant multiorgan donation program. Islets were used for research exclusively if they could not be used for clinical purposes and if research consent had been obtained according to national laws. Primary human islets were cultured in CMRL 1066 (Corning, Amsterdam, The Netherlands), 17.5 µg mL⁻^1^ Ciproxin, 1.05 mg mL⁻^1^ nicotinamide, 43.9 ng mL⁻^1^ gentamicin, 1.75 mM L‐Glutamin, 8.77 mM HEPES, 10% fetal calf serum in ultra‐low adherent plates (Corning, Amsterdam, The Netherlands). Donor information can be found in Table S1 (Supporting Information).

INS‐1E cells were cultured in RPMI, glutamax, 100 U mL^−1^ penicillin, 100 µg mL⁻^1^ streptomycin, 5% fetal calf serum, 1 mM Sodium Pyruvate, 10 mM HEPES, 50 µM β‐mercaptoethanol. INS1e cells were cultured in 12‐well plates at a cell density of 250.000 cells/well (Corning Costar, Amsterdam, The Netherlands). The cells were incubated at 37 °C in 5% CO2 in a humidified atmosphere. The number of experiments on islets from different donors or passages from cells is indicated in the figure legends.

### 2.3 In Vitro Treatments

Human islets and INS1E cells were treated with IL‐1β (1 ng mL⁻^1^; R&D systems, Dublin, Ireland) + IFNγ (50 ng mL⁻^1^; R&D systems, Dublin, Ireland), IFNα (1000 U mL⁻^1^; Merck, Amsterdam, The Netherlands), BMP2 (50 ng mL⁻^1^; R&D systems, Dublin, Ireland), BMP4 (50 ng mL⁻^1^; R&D systems, Dublin, Ireland) or LDN193189 (120 nM; Sigma Aldrich, Amsterdam, The Netherlands) for 2 to 72 h, as indicated in the figure legends. For the BMP2/4 experiments, the medium was refreshed every 24 h.

### 2.4 RNA Isolation and Real‐Time PCR

RNA of cell lysates was isolated according to the protocol of the RNeasy Micro kit (Qiagen, Venlo, The Netherlands). Subsequentially, the concentration and purity of the isolated RNA were measured (Thermo ScientificTM NanoDrop 1000), and reversed transcription was performed by the use of M‐MLV reverse transcriptase (Life Technologies, Bleiswijk, The Netherlands) and oligo(dT). For the quantitative PCR, 1.25  ng cDNA, SYBR GREEN PCR Master (Bio‐Rad, Hercules, USA), and 10 µM primer mix (forward and reverse) were used. Quantitative PCR was performed in a CFX384 Touch Real‐Time PCR Detection System (Bio‐Rad, Lunteren, The Netherlands). The list of primers used in this study can be found in Tables S2 and S3 (Supporting Information).

### 2.5 Glucose‐Stimulated Insulin Secretion

Human islets (≈30 islet equivalents (IEQ) per well) were seeded in a 96‐well transwell plate, and preincubated for 90 min in low‐glucose (2 mmol L⁻^1^) Krebs buffer (115 mmol L⁻^1^ NaCl, 5 mmol L⁻^1^ KCl, 24 mmol L⁻^1^ NaHCO_3_, 2.2 mmol L⁻^1^ CaCl_2_, 1 mmol L⁻^1^ Mgcl_2_, 20 mmol L⁻^1^ HEPES, and 0.2% human serum albumin) at pH 7.4. Islets were subsequently transferred to low (2 mmol L⁻^1^) glucose‐containing buffer for 60 min and then to high (20 mmol L⁻^1^) glucose‐containing buffer for 60 min. Glucose‐stimulated insulin secretion of INS‐1E cells was performed with the same protocol in 12‐well plates (Corning Costar, Amsterdam, The Netherlands). Insulin secretion was assessed using a human insulin ELISA kit (Mercodia, Huissen, The Netherlands) or a rat insulin ELISA kit (Mercodia, Huissen, The Netherlands). Insulin secretion was normalized to DNA content, which was assesed by Quant‐iT PicoGreen dsDNA assay kit (Thermo Fisher Scientific, Breda, The Netherlands).

### 2.6 Statistical Analysis

Statistical analysis was conducted using GraphPad Prism 9.3. One‐way ANOVA followed by Tukey's Multiple Comparison Test or paired Student T‐test was used to compare the group mean difference between cells cultured in different conditions (delta Ct calculated from *β‐Actin* (islets) or *Tbp* (INS‐1E) reference genes). Values are expressed as mean ± SEM and tests were considered significant when p < 0.05.

## 3 Results

### 3.1 Primary Human β‐Cells Express the Molecular Machinery for BMP Signaling

We first assessed the expression of the BMP signaling machinery in primary human β‐cells using a single‐cell RNAseq dataset of primary human islet cells generated in our lab. The main cell types were identified by expression of key genes (Figure 1a; Figure S1, Supporting Information). Of note, γ, endothelial, and acinar cells were not included in further analyses due to low cell numbers in the dataset (Figure 1; Table S4, Supporting Information).

Primary human β‐cells expressed both BMP receptors type 1 and type 2: *ACVR1* (type 1) and *BMPR2* (type 2) are the most highly expressed, as in all pancreatic cell types analyzed in this study (Figure 1b). The BMP mediator *SMAD5* was the most highly expressed in all cell types, while *SMAD9 (*also known as *SMAD8)* was mainly found in β‐cells (Figure 1c). Finally, the BMP target gene *ID1* was more predominant in β‐cells while *ID1‐4* was seen in all cell types (Figure 1d).

Collectively, these data show that primary human β‐cells express the molecular machinery required for proper BMP signaling.

### 3.2 BMP Signaling Is Enhanced in Primary Human Islets and β‐Cells upon Inflammation

We set out to investigate whether BMP signaling is altered in β‐cells upon inflammation. Single‐cell transcriptomics of primary human islets treated with pro‐inflammatory cytokines or left untreated, showed that the BMP target genes *ID1*, *ID3*, and *ID4* are upregulated in β‐cells upon IFNα as compared to the untreated control (p < 0.001) (Figure 2a–c) while IL‐1β+IFNγ did not significantly alter *ID*‐gene expression.

Activation of the BMP signaling pathway was further assessed by qPCR in human islets. *ID1, ID2*, and *ID4* genes were upregulated in human islets treated with IL‐1β+IFNγ for 24 h (1.9, 1.6, and 2.2‐fold respectively, p < 0.01 vs control) (Figure 2d), while *ID1* and *ID2* genes were upregulated upon treatment with IFNα for 24 h (1.7, and 1.3‐fold respectively, p < 0.05 vs control) (Figure 2e). Of note, ER‐stress genes *ATF3* and *CHOP* were found to be upregulated upon IL‐1β+IFNγ or IFNα, respectively, as expected (Figure S2a, Supporting Information). No striking differences were found in *ID*‐gene expression of islets exposed to IL‐1β+IFNγ or IFNα between 24 h and 72 h treatments (Figure S2b,c, Supporting Information). Importantly, the BMP antagonist LDN193189 inhibited the expression of *ID* genes, also in the presence of cytokines (Figure 2d,e).

Together, these data indicate that BMP signaling is enhanced in primary human β‐cells upon inflammation.

### 3.3 Distinct Pro‐Inflammatory Treatments Upregulate BMP2 or BMP4 Gene Expression

**Figure 3:**
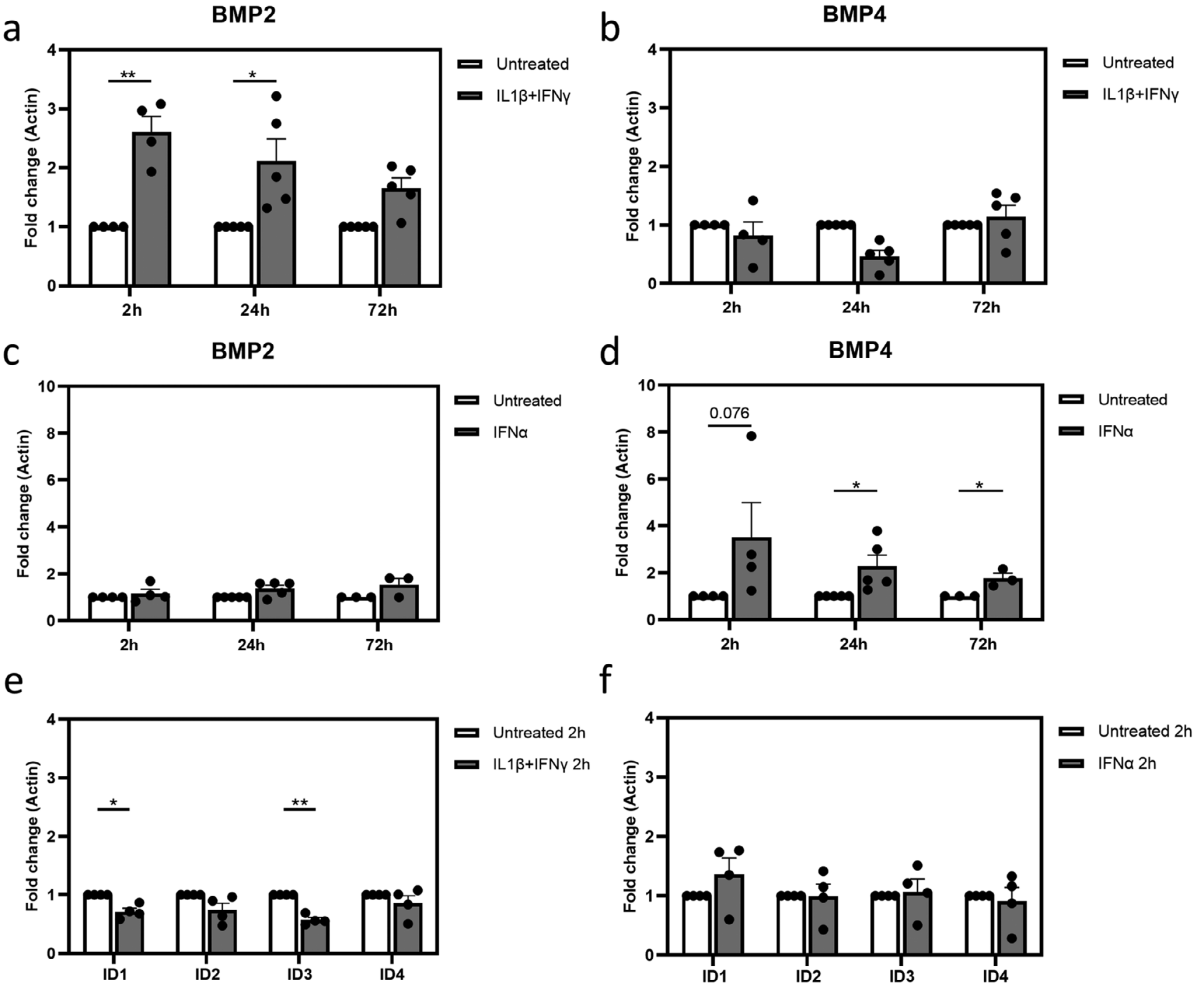
Inflammation activates *BMP2* and *BMP4* gene expression. a) *BMP2* expression in human islets (2 h n = 4, 24 h n = 5; 72 h n = 5) exposed for 2‐, 24‐ and 72 h to IL‐1β and IFNγ. b) *BMP4* expression in human islets (2 h n = 4, 24 h n = 5; 72 h n = 5) exposed for 2‐, 24‐ and 72 h to IL‐1β and IFNγ. c) *BMP2* expression in primary human islets exposed for 2‐, 24‐ and 72 h to IFNα (2h: n = 4, 24h: n = 5, 72h: n = 3). d) *BMP4* expression in primary human islets exposed for 2‐, 24‐ and 72 h to IFNα (2h: n = 4, 24h: n = 5, 72h: n = 3). e) *ID* expression in human islets (n = 4) exposed for 2 h to IL‐1β and IFNγ f) *ID* expression in islets exposed for 2 h to IFNα (n = 4). **p < 0.01, *p < 0.05.

Next, we assessed the gene expression levels of the ligands BMP2 and BMP4 under the same pro‐inflammatory conditions. *BMP2* gene expression was increased in human islets subjected to IL‐1β+IFNγ for 2 h and 24 h (2.6, 2.1‐fold respectively, p < 0.05 vs untreated) (**Figure** **3**a) while *BMP4* gene expression was not altered (Figure 3b). In contrast, human islets treated with IFNα did not show any change in *BMP2* expression (Figure 3c) while they showed an upregulation of the *BMP4* gene at 2 h, 24 h, and 72 h (3.5, 2.3, and 1.8‐fold respectively, p < 0.05 vs untreated) (Figure 3d). Of note, *BMP2* and *BMP4* were found to be expressed mainly in duct cells according to our scRNAseq dataset (Figure S2d, Supporting Information).

Interestingly, upregulation of *BMP2* was seen already at 2 h post‐IL‐1β+IFNγ treatment, while the BMP target gene expression was not yet increased (Figure 3e). The early induction of BMP2 upon IL‐1β+IFNγ could be mediating the upregulation of ID genes. Similarly, a trend of IFNα‐enhanced *BMP4* expression is seen after 2 h (3.5‐fold; p = 0.076, Figure 3d), while *ID* gene expression was unaffected (Figure 3f).

Taken together, this data indicates that the induction of BMP signaling could result from *BMP2* or *BMP4‐*mediated upregulation upon IL‐1β+IFNγ, and IFNα exposure, respectively.

### 3.4 Enhanced BMP Signaling Has a Negative Effect on β‐Cell Genes and Function, and ER Stress

We hypothesized that enhanced BMP signaling contributes to the detrimental effects of proinflammatory stress on β‐cells. Therefore, we treated INS1E cells with recombinant BMP2 (50 ng mL⁻^1^) or BMP4 (50 ng mL⁻^1^) for 72 h. *ID* gene expression was upregulated, validating the efficacy of the treatment (Figure S3a, Supporting Information).

**Figure 4:**
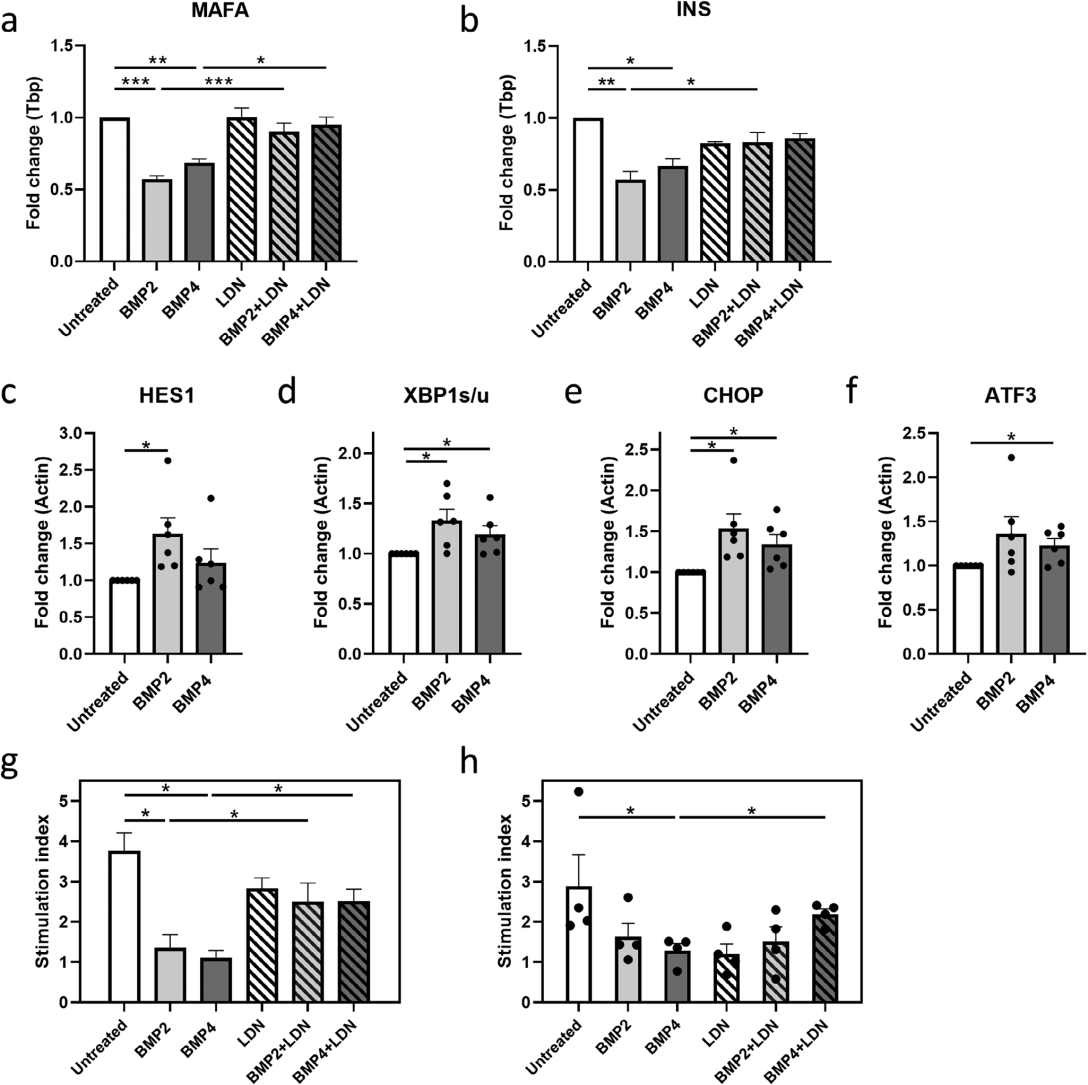
Enhanced BMP signaling has a negative effect on beta‐cell maturity, ER stress, and function. a) *MAFA*‐gene expression in INS‐1E cells treated with BMP2 (50 ng mL⁻^1^), BMP4 (50 ng mL⁻^1^), LDN (120 nM), or combination (n = 4). b) *INS*‐gene expression in INS‐1E cells treated with BMP2, BMP4, LDN, or combination (n = 4). Expression of c) *HES1* d) XBP1spliced/XBP1unspliced gene ratio e) *CHOP* f) *ATF3* in human islets treated with BMP2 or BMP4 (n = 6). g) Stimulation index of glucose‐stimulated insulin secretion in INS‐1E after 72 h treatment with BMP2, BMP4, or LDN (n = 3). h) Stimulation index of glucose‐stimulated insulin secretion from human islets after 72 h treatment with BMP2, BMP4, or LDN (n = 4). ***p < 0.001, **p < 0.01, *p < 0.05.

Upon β‐cell stress, *MAFA* expression is reduced prior to the reduction of other β‐cell markers, such as PDX1 and NKX6‐1.^[^ ^11^, ^35^, ^36^, ^37^ ^]^ We found a reduced expression of the β‐cell maturity genes insulin (*INS*) and *MAFA* for both BMP2 and BMP4 treatments (*MAFA*: 43%, and 32% reduction; *INS*: 43%, and 33% reduction respectively, p < 0.05 vs untreated) (**Figure** **4**a,b) with no effect on cell death (Figure S3b, Supporting Information). This downregulation was prevented by coincubation with LDN193189 (120 nM) (Figure 4a,b). LDN treatment alone did not alter *MAFA* or *INS* expression, indicating no effect of basal BMP signaling on β‐cell genes. A similar treatment with BMP2 and BMP4 performed on primary human islets shows a less pronounced upregulation of the *ID* genes, as compared to INS1E (Figure S3c, Supporting Information), and did not alter *MAFA* and *INS* expression (Figure S3d,e, Supporting Information). Nonetheless, an increase in *HES1* expression, which has been associated with β‐cell dedifferentiation, was found in BMP2‐treated islets (1.6‐fold, p < 0.05 vs untreated) (Figure 4c). This effect was associated with an increase in ER stress genes. The ratio of *spliced XBP1* vs. *unspliced XBP1*, and *CHOP* expression were upregulated upon BMP2 and BMP4 treatment (*XBP1s/u*: 1.3 and 1.2‐fold, *CHOP*: 1.5 and 1.2, for BMP2 and BMP4 respectively, p < 0.05 vs. untreated). In addition, *ATF3* was upregulated upon BMP4 treament (1.2‐fold, p < 0.05 vs. untreated) (Figure 4e,f).

Finally, we assessed β‐cell function, and found that treatment with recombinant BMP ligands triggered a strong reduction in glucose‐stimulated insulin secretion (GSIS) in both INS1E and human islets, without affecting basal insulin secretion (data not shown). BMP2 and BMP4 treatment of INS1E cells caused an 87% and 96% reduction in stimulation index (SI), respectively, compared to the untreated cells (p < 0.05 vs untreated) (Figure 4g). Treatment with BMP2 and BMP4 of human islets decreased SI with 34% (n.s.) and 85% respectively (p < 0.05 vs untreated) (Figure 4h). Interestingly, a trend toward reduction of insulin secretion upon high glucose stimulation was observed in LDN treated islets, indicating that inhibition of basal BMP signaling lowers GSIS.

Collectively, our data reveal that BMP2/BMP4 induced BMP signaling triggers detrimental effects on β‐cell genes and function.

## 4 Discussion

The molecular mechanisms underlying inflammation induced β‐cell failure in the development of type 1 and type 2 diabetes has been the focus of extensive research.^[^ ^2^, ^3^, ^4^, ^5^, ^6^, ^7^ ^]^ In this study, we demonstrate that pro‐inflammatory conditions activate BMP signaling in primary human islets, leading to altered β‐cell identity, increased ER stress and reduced β‐cell function. Therefore, we propose that enhanced BMP signaling contributes to the detrimental effect of inflammation‐induced β‐cell failure in diabetes.

Earlier mouse studies showed contrasting results. Some reports are in line with our findings: ID1 inhibition has been shown to improve glucose homeostasis in high‐fat diet mice.^[^ ^38^ ^]^ Treatment with the BMP pathway inhibitor, noggin, reduced serum glucose levels in db/db mice.^[^ ^39^ ^]^ However, transgenic overexpression of BMP4 or treatment with recombinant BMP4 enhanced insulin secretion upon high glucose stimulation.^[^ ^26^ ^]^ Besides, impaired insulin secretion was found in mice with specific knockout of BMP4 in pancreatic pericytes,^[^ ^40^ ^]^ altogether suggesting a multi‐factored and complicated role of BMP on β‐cell function.

We show that distinct BMP ligands are upregulated by different pro‐inflammatory conditions. BMP2 ligand is induced in human islets subjected to IL‐1β+IFNγ treatment, as reported earlier after 48 h of IL‐1β (2 ng mL⁻^1^), IFN‐γ (5 ng mL⁻^1^), and TNF‐α (10 ng mL⁻^1^) treatment,^[^ ^16^ ^]^ but not upon IFNα. On the other hand, we found a specific increase in BMP4, but not BMP2, expression upon IFNα. The distinct effect of IFNγ versus IFNα on β‐cells has been reported recently.^[^ ^41^ ^]^ Altogether these findings point toward a role for BMP signaling in both early (IFNα‐mediated inflammation) and late (IL‐1β+IFNγ‐mediated inflammation) stages during the development of diabetes.

We found that activation of BMP signaling through recombinant BMP2 or BMP4 induces β‐cell identity changes, as evidenced by reduced *INS* and *MAFA* expression in INS1E, and increased *HES1* gene expression in human islets. Supporting our findings, decreased expression of β‐cell marker *UCN3* and increased expression of *HES1* have been found in mouse islets upon BMP2 treatment for 10 days.^[^ ^24^ ^]^ Increased *HES1* expression is linked with β‐cell dedifferentiation in human islets^[^ ^42^ ^]^ and has been reported to bind to NeuroD1 and PDX1, thereby inhibiting insulin synthesis.^[^ ^43^ ^]^ Correspondingly, we showed reduced *INS* gene expression and impaired glucose‐stimulated insulin secretion in β‐cells upon BMP2 or BMP4 treatment. These findings partly echo earlier studies,^[^ ^30^ ^]^ altogether further strengthening the causative link between enhanced BMP signaling and β‐cell dysfunction.

In addition, we have emerging evidence indicating that enhancing BMP signaling triggers an ER stress response in human islets. BMP2‐induced ER stress has been found in human coronary artery smooth muscle cells, myoblasts, and osteoblasts upon higher concentrations of BMP2 (100–200 ng mL⁻^1^).^[^ ^44^, ^45^, ^46^ ^]^ Initiation of ER stress has also been reported by BMP4 treatment of myeloma and B‐cell hybridoma cells, as a mechanism of apoptosis induction.^[^ ^47^ ^]^ We previously reported that ER stress causes β‐cell identity loss and reduces glucose stimulated insulin secretion in human pancreatic islets.^[^ ^36^ ^]^ Furthermore, ER stress has been linked to β‐cell dysfunction in type 1 diabetes,^[^ ^48^, ^49^, ^50^ ^]^ as well as in type 2 diabetes.^[^ ^51^, ^52^ ^]^

## 5 Conclusions

Altogether, these data indicate that inhibition of BMP‐signaling (or downstream genes) could be a target to preserve β‐cell identity and function in a pro‐inflammatory environment. In light of current research focusing on preservation of a functional β‐cell mass, this study calls for further investigating into the role of the BMP/SMAD signaling pathway during the development of diabetes. The study on small molecules modulating the activity of BMP receptors is of high interest and showed promising safety outcomes in the context of different types of cancer, pulmonary arterial hypertension and osteoporosis.^[^ ^53^ ^]^ Despite the involvement of the BMP pathway in the development and maintenance of many organs, no major side effects or toxicity were detected during systemic BMP inhibition in mice models of osteochondroma formation, fibrodysplasia ossificans progressiva, anemia of inflammation, atherosclerosis and vascular calcifications, and prostate cancer induced bone disease.^[^ ^54^, ^55^, ^56^, ^57^, ^58^ ^]^ Therefore, BMP inhibition appears as a potential target for intervention in the context of diabetes as well. Future research should be focused on deciphering the specific mechanisms by which BMP can affect insulin secretion or identity loss in β‐cells and whether modulation of BMP signaling might be beneficial in the context of diabetes.

## Conflict of Interest

The authors declare no conflict of interest.

## Author contributions

ED designed and performed the experiments and wrote the manuscript. NdG provided technical support. JT participated in the conception of the idea and hypothesis of the project. AM performed scRNAseq data analysis. FC and EdK supervised the project. FC acquired the funding for the project and wrote the manuscript. All authors contributed to the article and approved the final version.

## Supporting information



Supporting Information

## Data Availability

The data that support the findings of this study are available from the corresponding author upon reasonable request.
