# Supplementary material for: Enhanced BMP Signaling Alters Human β‐Cell Identity and Function
Source: Adv Biol (Weinh). 2024 Nov 5;9(1):2400470. doi: 10.1002/adbi.202400470 (PMC11760635; doi:10.1002/adbi.202400470)
Supplement: Supplementary file 1 — Supporting Information [file ADBI-9-2400470-s001.docx]

**Figure S1**

**CD24**

**KRT19**

**PRSS1**

**COL1A1**

**ESAM**


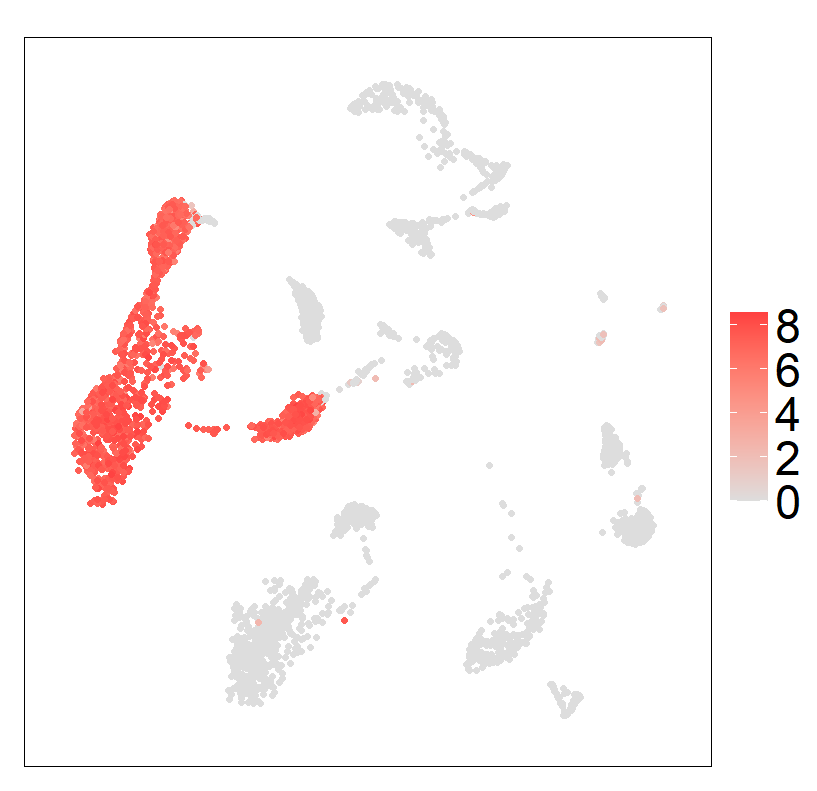

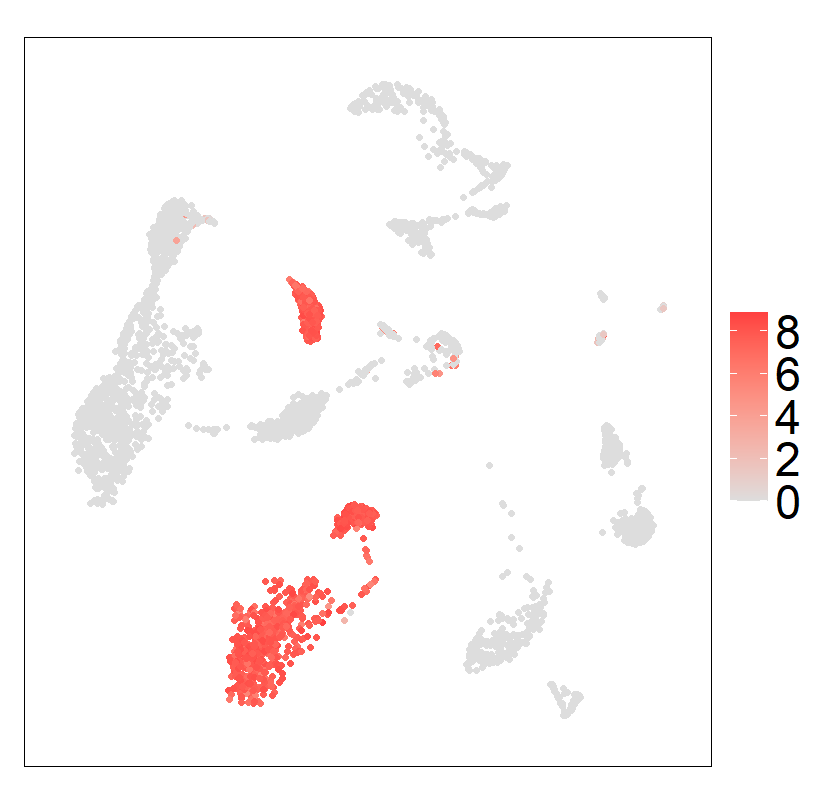

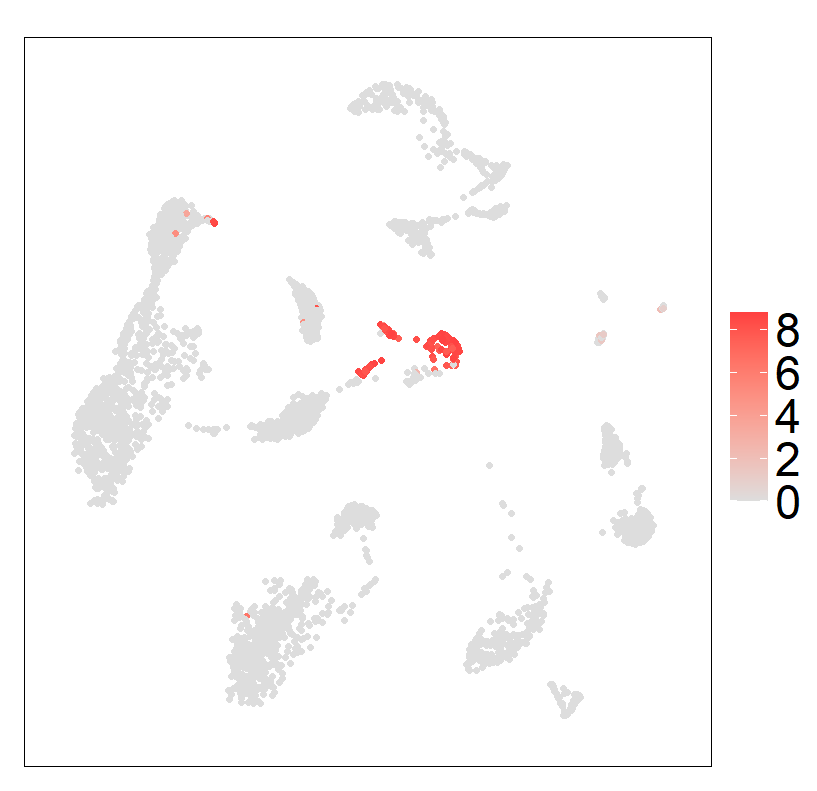

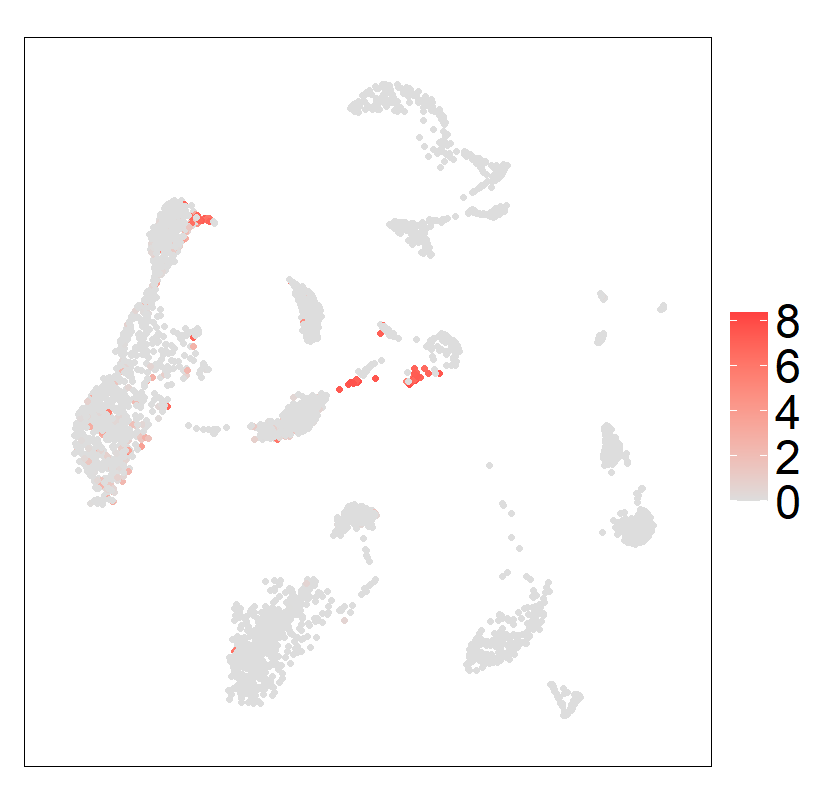

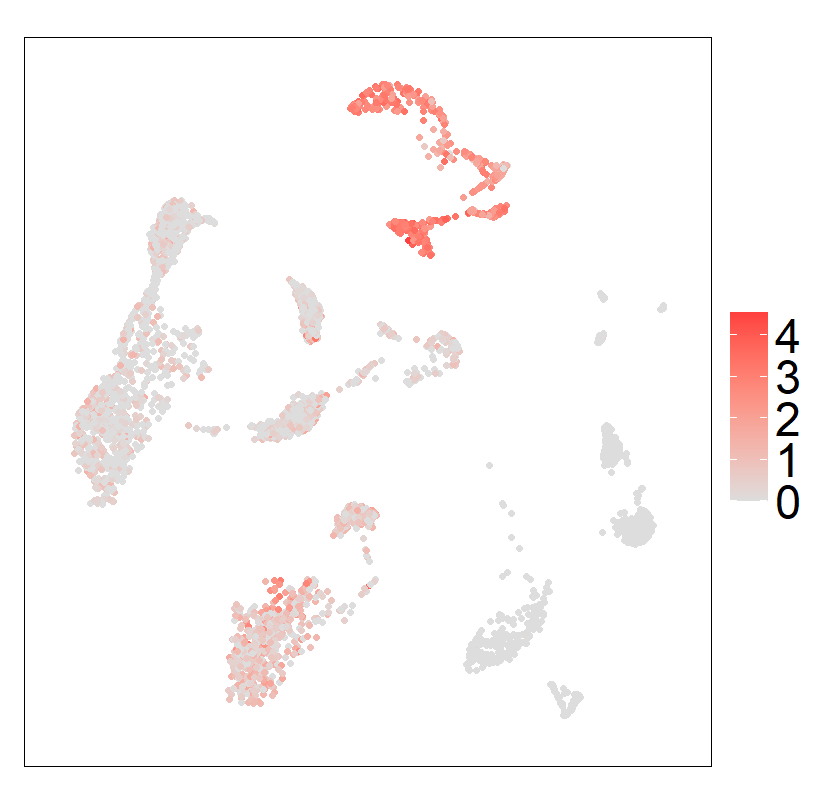

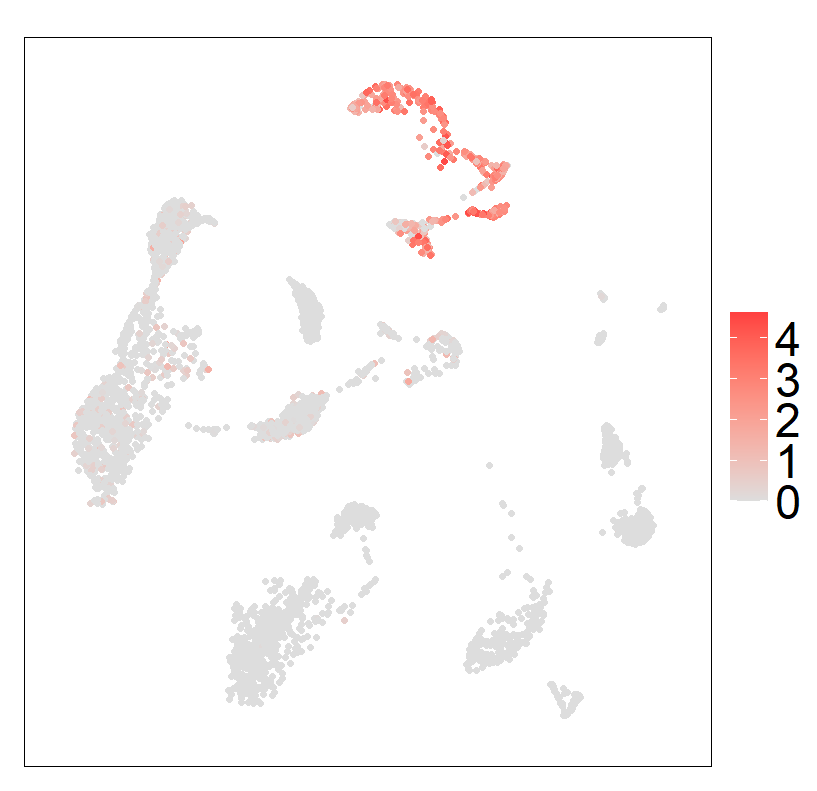

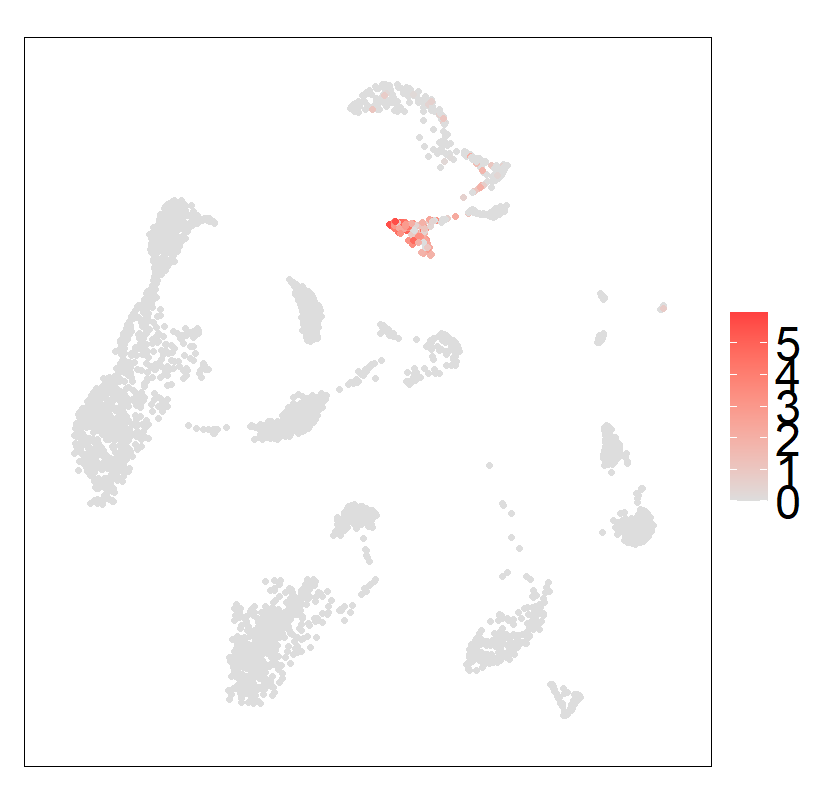

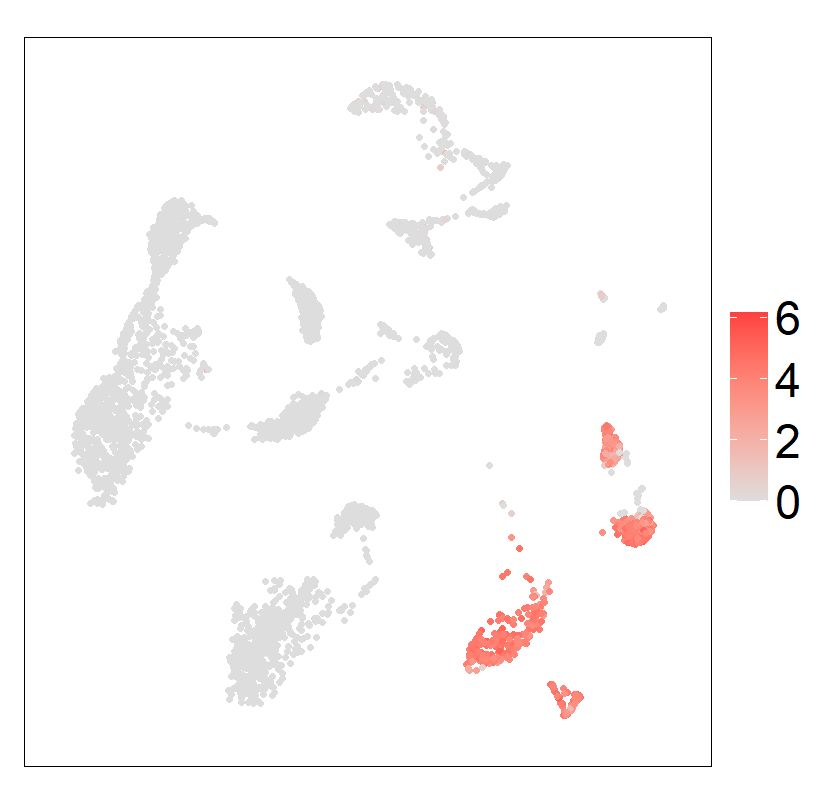

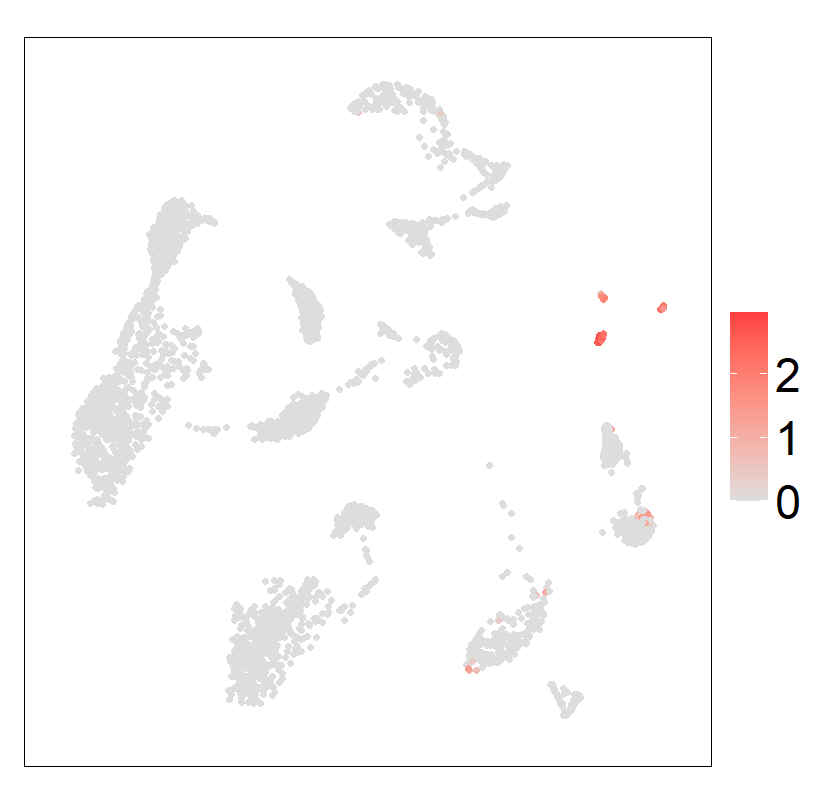


**GCG**

**INS**

**SST**

**PPY**

**Figure S1.** UMAPs representing expression of the canonical identity markers of each pancreatic cell type: glucagon (*GCG*), insulin (*INS*), somatostatin (*SST*), pancreatic polypeptide (*PPY*), cluster of differentiation 24 (*CD24*), keratin 19 (*KRT19*), trypsin 1 (*PRSS1*), collagen type I alpha 1 chain (*COL1A1*), and endothelial cell adhesion molecule (*ESAM*).

a

**Figure S2**

c

d

b


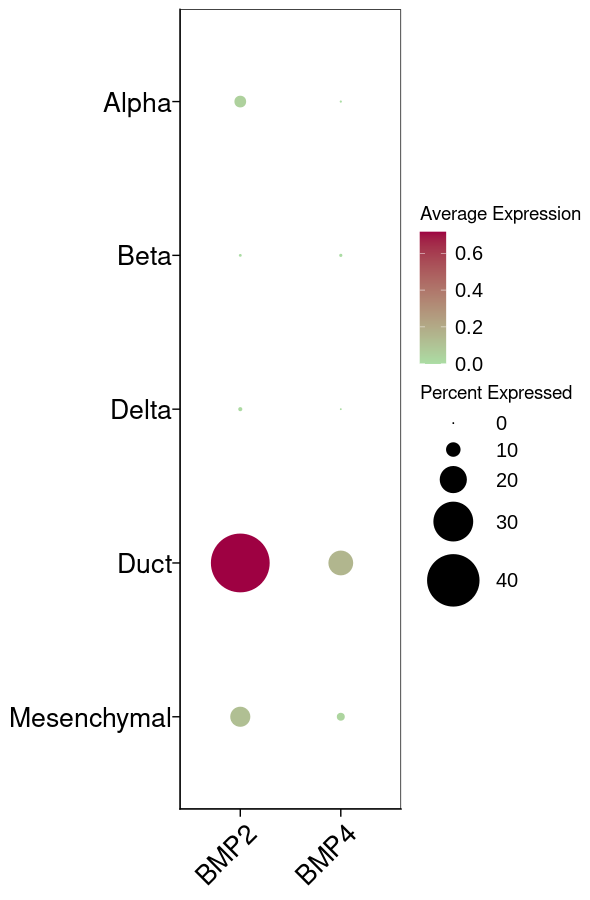


**Figure S2**. BMP signaling is active in human pancreatic islets untreated or treated with stressors for 24 h or 72 h. a: ER-stress genes (*ATF3, CHOP, XBP1s/u*) expression in islets treated with IL-1β+IFNγ, IFNα or untreated for 24 h (n = 5). b: *ID*-gene expression in primary human islets after 24- and 72 h treatment with IL-1β and IFNγ or untreated (n = 5). c: *ID*-gene expression in primary human islets after 24- and 72 h treatment with IFNα or untreated (24 h: n = 5, 72 h: n = 3). d: Expression of *BMP2* and *BMP4* genes in different cell-type clusters. ***p<0.001, **p<0.01, *p<0.05.

**Figure S3**

a

c

d

e


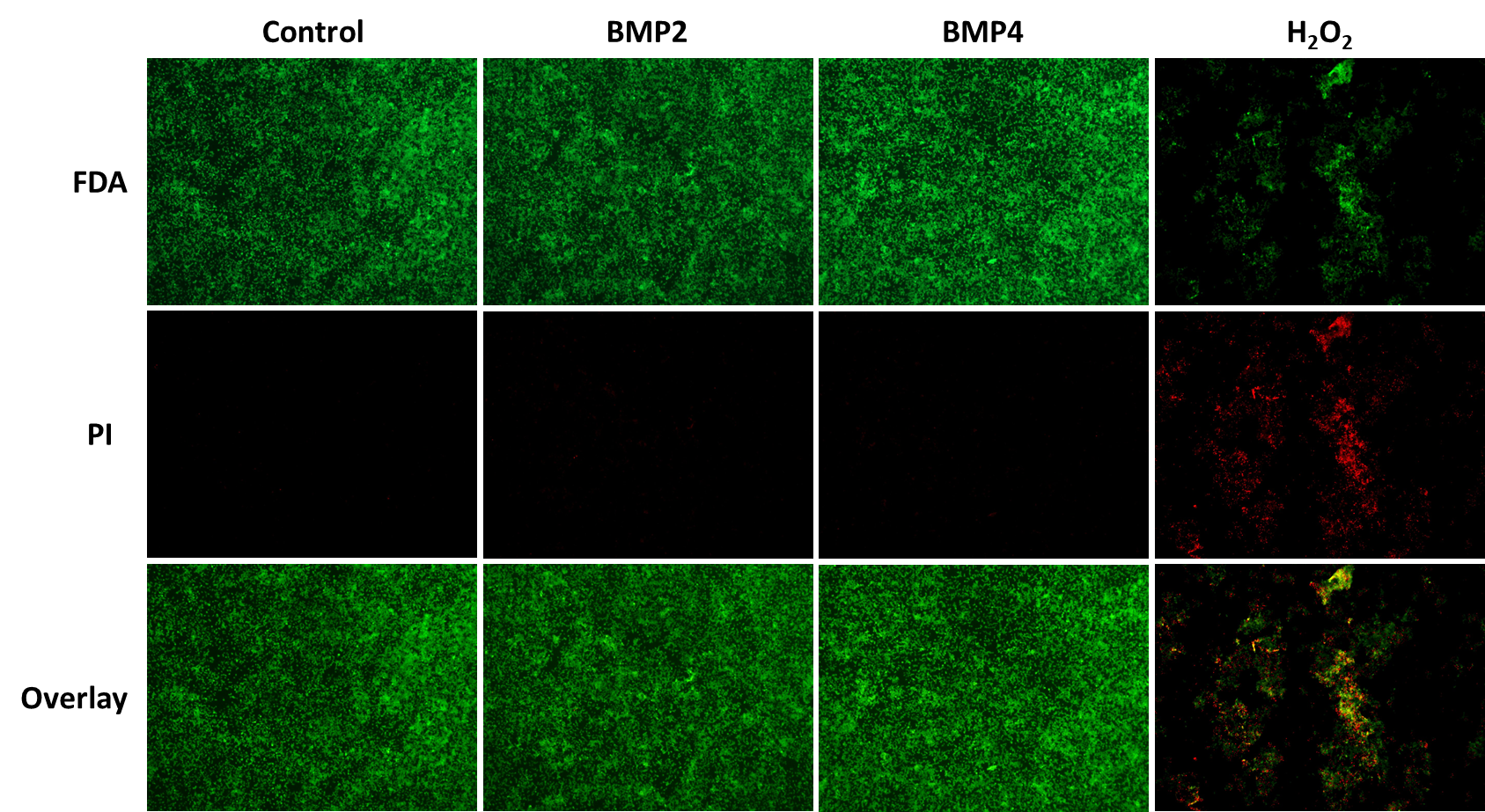


b

**Figure S3.** BMP2 and BMP4 treatment upregulate BMP-target genes. a: *ID-*gene expression in untreated INS-1E or INS-1E exposed for 72 h to BMP2 (50 ng/ml), BMP4 (50 ng/ml) or LDN (120 nM) (n = 4). b: FDA (viable, green) and PI (dead, red) staining of INS-1E cells treated for 72 h with BMP2 (50 ng/ml), BMP4 (50 ng/ml), 90 minutes with H_2_O_2_ (500 µM) or untreated. Expression in untreated human islets or human islets exposed for 72 h to BMP2 (50 ng/ml), BMP4 (50 ng/ml) or LDN (120 nM) of c: *ID*-genes. d: *MAFA*. e: *INS* (n = 6). ****p<0.0001, ***p<0.001, **p<0.01, *p<0.05.

**Table S1:** Human islet checklist

**Checklist for reporting human islet preparations used in research**

Adapted from Hart NJ, Powers AC (2018) Progress, challenges, and suggestions for using human islets to understand islet biology and human diabetes. Diabetologia <https://doi.org/10.1007/s00125-018-4772-2>

| **Islet preparation** | **1** | **2** | **3** | **4** | **5** | **6** | **7** | **8** |
| --- | --- | --- | --- | --- | --- | --- | --- | --- |
| **MANDATORY INFORMATION** | | | | | | | | |
| Unique identifier | R249 | R250 | P671 | P669 | P721 | R233 | R227 | P699 |
| Donor age (years) | 70 | 47 | 57 | 55 | 46 | 57 | 57 | 52 |
| Donor sex (M/F) | M | M | M | M | M | F | M | M |
| Donor BMI (kg/m^2^) | 27 | 27 | 25 | 26 | 30 | 35 | 24 | 25 |
| Donor HbA_1c_ or other measure of blood glucose control | 5.6 | 5.4 | 5.5 |  | 5.9 | 6.3 | 5.4 |  |
| Origin/source of islets^b^ |  |  |  |  |  |  |  |  |
| Islet isolation centre | LUMC | LUMC | LUMC | LUMC | LUMC | LUMC | LUMC | LUMC |
| Donor history of diabetes? Please select yes/no from drop down list | No | No | No | No | No | No | No | No |
| **If Yes, complete the next two lines if this information is available** | | | | | | | | |
| Diabetes duration (years) |  |  |  |  |  |  |  |  |
| Glucose-lowering therapy at time of death^c^ |  |  |  |  |  |  |  |  |
| **RECOMMENDED INFORMATION** | | | | | | | | |
| Donor cause of death | Cerebral Ischemia | Cardiac Arrest | Suicide | Cerebral Ischemia | Circulational | Sub Arachnoidal Bleeding | Euthanasia | Sub Arachnoidal Bleeding |
| Warm ischaemia time (h) |  |  |  |  |  |  |  |  |
| Cold ischaemia time (h) |  |  |  |  |  |  |  |  |
| Estimated purity (%) |  |  |  |  |  |  |  |  |
| Estimated viability (%) |  |  |  |  |  |  |  |  |
| Total culture time (h)^d^ |  |  |  |  |  |  |  |  |
| Glucose-stimulated insulin secretion or other functional measurement^e^ |  |  |  |  |  |  |  |  |
| Handpicked to purity? Please select yes/no from drop down list |  |  |  |  |  |  |  |  |
| Additional notes |  |  |  |  |  |  |  |  |

| **Islet preparation** | **9** | **10** | **11** | **12** | **13** | **14** | **15** | **16** |
| --- | --- | --- | --- | --- | --- | --- | --- | --- |
| **MANDATORY INFORMATION** | | | | | | | | |
| Unique identifier | R225 | P689 | R230 | R234 | P652 | R189 | P655 | R186 |
| Donor age (years) | 71 | 55 | 51 | 73 | 49 | 78 | 46 | 70 |
| Donor sex (M/F) | M | M | F | M | M | F | F | M |
| Donor BMI (kg/m^2^) | 25 | 32 | 35 | 26 | 26 | 21 | 32 | 25 |
| Donor HbA_1c_ or other measure of blood glucose control | 5.7 | 5.5 |  | 6.45 | 5.4 | 5.9 |  | 5.6 |
| Origin/source of islets^b^ |  |  |  |  |  |  |  |  |
| Islet isolation centre | LUMC | LUMC | LUMC | LUMC | LUMC | LUMC | LUMC | LUMC |
| Donor history of diabetes? Please select yes/no from drop down list | No | No | No | No | No | No | No | No |
| **If Yes, complete the next two lines if this information is available** | | | | | | | | |
| Diabetes duration (years) |  |  |  |  |  |  |  |  |
| Glucose-lowering therapy at time of death^c^ |  |  |  |  |  |  |  |  |
| **RECOMMENDED INFORMATION** | | | | | | | | |
| Donor cause of death | Cerebro Vascular Accident | Sub Arachnoidal Bleeding | Sub Arachnoidal Bleeding | Cerebral Ischemia | Trauma: Capitis | Sub Arachnoidal Bleeding | Intra Cerebral Bleeding | Sub Arachnoidal Bleeding |
| Warm ischaemia time (h) |  |  |  |  |  |  |  |  |
| Cold ischaemia time (h) |  |  |  |  |  |  |  |  |
| Estimated purity (%) |  |  |  |  |  |  |  |  |
| Estimated viability (%) |  |  |  |  |  |  |  |  |
| Total culture time (h)^d^ |  |  |  |  |  |  |  |  |
| Glucose-stimulated insulin secretion or other functional measurement^e^ |  |  |  |  |  |  |  |  |
| Handpicked to purity? Please select yes/no from drop down list |  |  |  |  |  |  |  |  |
| Additional notes |  |  |  |  |  |  |  |  |

| **Islet preparation** | **17** | **18** | **19** | **20** | **21** | **22** |
| --- | --- | --- | --- | --- | --- | --- |
| **MANDATORY INFORMATION** | | | | | | |
| Unique identifier | R190 | R196 | R197 | R200 | R251 | R229 |
| Donor age (years) | 70 | 69 | 73 | 69 | 54 | 54 |
| Donor sex (M/F) | M | M | M | M | M | M |
| Donor BMI (kg/m^2^) | 24 | 22 | 25 | 24 | 24 | 34 |
| Donor HbA_1c_ or other measure of blood glucose control | 5.6 |  | 5.5 |  |  |  |
| Origin/source of islets^b^ |  |  |  |  |  |  |
| Islet isolation centre | LUMC | LUMC | LUMC | LUMC | LUMC | LUMC |
| Donor history of diabetes? Please select yes/no from drop down list | No | No | No | No | No | No |
| Diabetes duration (years) |  |  |  |  |  |  |
| Glucose-lowering therapy at time of death^c^ |  |  |  |  |  |  |
| **RECOMMENDED INFORMATION** | | | | | | |
| Donor cause of death | Suicide | Intra Cerebral Bleeding | Intra Cerebral Bleeding | Intra Cerebral Bleeding | Respirational | Trauma: Capitis |
| Warm ischaemia time (h) |  |  |  |  |  |  |
| Cold ischaemia time (h) |  |  |  |  |  |  |
| Estimated purity (%) |  |  |  |  |  |  |
| Estimated viability (%) |  |  |  |  |  |  |
| Total culture time (h)^d^ |  |  |  |  |  |  |
| Glucose-stimulated insulin secretion or other functional measurement^e^ |  |  |  |  |  |  |
| Handpicked to purity? Please select yes/no from drop down list |  |  |  |  |  |  |
| Additional notes |  |  |  |  |  |  |

**Table S2**: List of human primers

| Gene name | Sequence primer FW (5'→3') | Sequence primer RV (5'→3') | Source |
| --- | --- | --- | --- |
| *ACTIN* | TGCGTGACATTAAGGAGAAG | TGAAGGTAGTTTCGTGGATG | (1) |
| *ATF3* | GTGCCGAAACAAGAAGAAGG | TCTGAGCCTTCAGTTCAGCA | (2) |
| *BMP2* | GGAACGGACATTCGGTCCTT | CACCATGGTCGACCTTTAGGA | (3) |
| *BMP4* | CGTCCAAGCTATCTCGAGCC | CGGAATGGCTCCATAGGTCC | (4) |
| *CHOP* | GACCTGCAAGAGGTCCTGTC | CTCCTCCTCAGTCAGCCAAG | (5) |
| *HES1* | CCAAAGACAGCATCTGAGCA | CATTGATCTGGGTCATGCAG | (6) |
| *ID1* | CTGCTCTACGACATGAACGG | GAAGGTCCCTGATGTAGTCGAT | (7) |
| *ID2* | ATGAAAGCCTTCAGTCCCGT | TTCCATCTTGCTCACCTTCTT | (8) |
| *ID3* | CACCTCCAGAACGCAGGTGCTG | AGGGCGAAGTTGGGGCCCAT | (9) |
| *ID4* | TGAACAAGCAGGGCGACA | CGTGCAAAGAAAGAATGAAAG | (8) |
| *INS* | AAGAGGCCATCAAGCAGATCA | CAGGAGGCGCATCCACA | (10) |
| *MAFA* | CAGTCCTGCCGCTTCAAG | ACAGGTCCCGCTCTTTGG | (11) |
| *PDX1* | CCATGGATGAAGTCTACCAAAGCT | CGTGAGATGTACTTGTTGAATAGGAACT | (12) |
| *XBP1s* | CTGAGTCCGCAGCAGGTG | GAGATGTTCTGGAGGGGTGA | (5) |
| *XBP1u* | GGAGTTAAGACAGCGCTTGG | CTGCAGAGGTGCACGTAGTC | (13) |

**Table S3**: List of rat primers

| Gene name | Sequence primer FW (5'→3') | Sequence primer RV (5'→3') | Source |
| --- | --- | --- | --- |
| *ID1* | GAACCGCAAAGTGAGCAAGG | GGAACACATGCCGCCTCG | (14) |
| *ID2* | AGCATCCCCCAGAACAAGAAG | GGTGCAGCGTGACGATAGTG | (15) |
| *ID3* | GCATCTCCCGATCCAGACAG | CGGGTCAGTGGCAAAAACTC | (15) |
| *ID4* | GAGCATTGGCGACGTTGTTT | GAGAAAAAGTTCCCCGCCCT | (16) |
| *INS* | ATCCTCTGGGAGCCCCGC | AGAGAGCTTCCACCAAG | (17) |
| *MAFA* | CGCACCCGACTTCTTTCTGT | CTCAGAGTCCGAACCGAGG | (18) |
| *TBP* | TAAGGCTGGAAGGCCTTGTG | TCTGCTCTAACTTTAGCACCTGT | (15,19) |

**References**

1. Driehuis E, Kolders S, Spelier S, Lõhmussaar K, Willems SM, Devriese LA, et al. Oral Mucosal Organoids as a Potential Platform for Personalized Cancer Therapy. Cancer Discov. 2019;9(7):852-71.
2. Wang L, Liu Y, Du T, Yang H, Lei L, Guo M, et al. ATF3 promotes erastin-induced ferroptosis by suppressing system Xc(). Cell Death Differ. 2020;27(2):662-75.
3. Cheung VC, Peng CY, Marinić M, Sakabe NJ, Aneas I, Lynch VJ, et al. Pluripotent stem cell-derived endometrial stromal fibroblasts in a cyclic, hormone-responsive, coculture model of human decidua. Cell Rep. 2021;35(7):109138.
4. Yuan Z, Memarzadeh K, Stephen AS, Allaker RP, Brown RA, Huang J. Development of a 3D Collagen Model for the In Vitro Evaluation of Magnetic-assisted Osteogenesis. Sci Rep. 2018;8(1):16270.
5. Kracht MJL, de Koning EJP, Hoeben RC, Roep BO, Zaldumbide A. Bioluminescent reporter assay for monitoring ER stress in human beta cells. Sci Rep. 2018;8(1):17738.
6. Lee SH, Nam HJ, Kang HJ, Kwon HW, Lim YC. Epigallocatechin-3-gallate attenuates head and neck cancer stem cell traits through suppression of Notch pathway. Eur J Cancer. 2013;49(15):3210-8.
7. Zhao Y, Zhu J, Shi B, Wang X, Lu Q, Li C, et al. The transcription factor LEF1 promotes tumorigenicity and activates the TGF-β signaling pathway in esophageal squamous cell carcinoma. J Exp Clin Cancer Res. 2019;38(1):304.
8. Jung JW, Shim SY, Lee DK, Kwiatkowski W, Choe S. An Activin A/BMP2 chimera, AB215, blocks estrogen signaling via induction of ID proteins in breast cancer cells. BMC Cancer. 2014;14(1):549.
9. Ren J, Wang Y, Ware T, Iaria J, Ten Dijke P, Zhu HJ. Reactivation of BMP signaling by suboptimal concentrations of MEK inhibitor and FK506 reduces organ-specific breast cancer metastasis. Cancer Lett. 2020;493:41-54.
10. Ma H, Jeppesen JF, Jaenisch R. Human T Cells Expressing a CD19 CAR-T Receptor Provide Insights into Mechanisms of Human CD19-Positive β Cell Destruction. Cell Rep Med. 2020;1(6):100097.
11. Triñanes J, Ten Dijke P, Groen N, Hanegraaf M, Porrini E, Rodriguez-Rodriguez AE, et al. Tacrolimus-Induced BMP/SMAD Signaling Associates With Metabolic Stress-Activated FOXO1 to Trigger β-Cell Failure. Diabetes. 2020;69(2):193-204.
12. Szymczak F, Cohen-Fultheim R, Thomaidou S, de Brachène AC, Castela A, Colli M, et al. ADAR1-dependent editing regulates human β cell transcriptome diversity during inflammation. Front Endocrinol (Lausanne). 2022;13:1058345.
13. Groen N, Leenders F, Mahfouz A, Munoz-Garcia A, Muraro MJ, de Graaf N, et al. Single-Cell Transcriptomics Links Loss of Human Pancreatic β-Cell Identity to ER Stress. Cells. 2021;10(12).
14. Meng L, Teng X, Liu Y, Yang C, Wang S, Yuan W, et al. Vital Roles of Gremlin-1 in Pulmonary Arterial Hypertension Induced by Systemic-to-Pulmonary Shunts. J Am Heart Assoc. 2020;9(15):e016586.
15. [Available from: https://www.ncbi.nlm.nih.gov/tools/primer-blast/.
16. Albano F, Tucci V, Blackshear PJ, Reale C, Roberto L, Russo F, et al. ZFP36L2 Role in Thyroid Functionality. Int J Mol Sci. 2021;22(17).
17. Prause M, Christensen DP, Billestrup N, Mandrup-Poulsen T. JNK1 protects against glucolipotoxicity-mediated beta-cell apoptosis. PLoS One. 2014;9(1):e87067.
18. Wu H, Liu Y, Wang H, Xu X. High-fat diet induced insulin resistance in pregnant rats through pancreatic pax6 signaling pathway. Int J Clin Exp Pathol. 2015;8(5):5196-202.
19. Gambarotta G, Ronchi G, Friard O, Galletta P, Perroteau I, Geuna S. Identification and validation of suitable housekeeping genes for normalizing quantitative real-time PCR assays in injured peripheral nerves. PLoS One. 2014;9(8):e105601.

**Table S4**: Cell cluster information

| Treatment | Amount | Total_cells | Percent | Cluster |
| --- | --- | --- | --- | --- |
| Untreated | 591 | 1673 | 35.3257621 | Alpha |
| IL1β+IFNγ | 609 | 1673 | 36.40167364 | Alpha |
| IFNα | 473 | 1673 | 28.27256426 | Alpha |
| Untreated | 381 | 1061 | 35.90951932 | Beta |
| IL1β+IFNγ | 439 | 1061 | 41.37606032 | Beta |
| IFNα | 241 | 1061 | 22.71442036 | Beta |
| Untreated | 57 | 204 | 27.94117647 | Delta |
| IL1β+IFNγ | 89 | 204 | 43.62745098 | Delta |
| IFNα | 58 | 204 | 28.43137255 | Delta |
| Untreated | 20 | 22 | 90.90909091 | Gamma |
| IL1β+IFNγ | 1 | 22 | 4.545454545 | Gamma |
| IFNα | 1 | 22 | 4.545454545 | Gamma |
| Untreated | 160 | 398 | 40.20100503 | Duct |
| IL1β+IFNγ | 142 | 398 | 35.67839196 | Duct |
| IFNα | 96 | 398 | 24.12060302 | Duct |
| Untreated | 53 | 101 | 52.47524752 | Acinar |
| IL1β+IFNγ | 23 | 101 | 22.77227723 | Acinar |
| IFNα | 25 | 101 | 24.75247525 | Acinar |
| Untreated | 229 | 708 | 32.34463277 | Mesenchymal |
| IL1β+IFNγ | 220 | 708 | 31.07344633 | Mesenchymal |
| IFNα | 259 | 708 | 36.5819209 | Mesenchymal |
| Untreated | 26 | 75 | 34.66666667 | Endothelial |
| IL1β+IFNγ | 22 | 75 | 29.33333333 | Endothelial |
| IFNα | 27 | 75 | 36 | Endothelial |
